# Latest Advances in the Development of Eukaryotic Vaults as Targeted Drug Delivery Systems

**DOI:** 10.3390/pharmaceutics11070300

**Published:** 2019-06-28

**Authors:** Amanda Muñoz-Juan, Aida Carreño, Rosa Mendoza, José L. Corchero

**Affiliations:** 1 Institut de Biotecnologia i de Biomedicina, Universitat Autònoma de Barcelona, 08193 Bellaterra, Spain; 2 Networking Center on Bioengineering, Biomaterials and Nanomedicine (CIBER-BBN), 28029 Madrid, Spain; 3 Department of Genetics and Microbiology, Universitat Autònoma de Barcelona, 08193 Bellaterra, Spain

**Keywords:** eukaryotic vaults, nanoparticle, drug delivery systems, nanocage, protein self-assembly

## Abstract

The use of smart drug delivery systems (DDSs) is one of the most promising approaches to overcome some of the drawbacks of drug-based therapies, such as improper biodistribution and lack of specific targeting. Some of the most attractive candidates as DDSs are naturally occurring, self-assembling protein nanoparticles, such as viruses, virus-like particles, ferritin cages, bacterial microcompartments, or eukaryotic vaults. Vaults are large ribonucleoprotein nanoparticles present in almost all eukaryotic cells. Expression in different cell factories of recombinant versions of the “major vault protein” (MVP) results in the production of recombinant vaults indistinguishable from native counterparts. Such recombinant vaults can encapsulate virtually any cargo protein, and they can be specifically targeted by engineering the C-terminus of MVP monomer. These properties, together with nanometric size, a lumen large enough to accommodate cargo molecules, biodegradability, biocompatibility and no immunogenicity, has raised the interest in vaults as smart DDSs. In this work we provide an overview of eukaryotic vaults as a new, self-assembling protein-based DDS, focusing in the latest advances in the production and purification of this platform, its application in nanomedicine, and the current preclinical and clinical assays going on based on this nanovehicle.

## 1. Introduction

Nanomedicine is a translational science whose objective is to obtain new therapies and diagnostic tools using the new available capabilities of nanotechnology [1]. It is applied in drug delivery, diagnosis, imaging, and therapy fields. The application of nanotechnology in the design of new drug delivery systems (DDSs) is, at this moment, one of the most active fields in nanomedicine research. 

Conventional drug administration regimes use large amounts of the active principle, resulting in high costs, undesired side-effects and low therapeutic efficacy because only small amounts of the drug finally reach the target cells or tissues [2,3]. DDSs based on nanoparticles (NPs) have made a remarkable difference in site-specific release of chemotherapeutic agents, due to their physical and chemical characteristics and biological attributes. The use of such nanocarriers improves drug biodistribution, targeting active molecules to diseased cells and tissues while protecting healthy ones.

Several NPs types are being extensively explored, including polymeric micelles [4], solid nanoparticles [5], solid lipid nanoparticles (SLN) [6], nanostructured lipid carriers (NLC) [7], liposomes [8], inorganic nanoparticles [9], dendrimers [10], or magnetic nanoparticles [11]. Among them, biopolymer-based nanoparticles, including protein nanoparticles, are actively explored and used in pharmaceuticals [12,13].

Protein nano-DDSs are protein structures formed by the assembly of multiple copies of one or several different proteins [14]. Nature offers such functional macromolecular structures that can be easily manipulated for nanobiotechnology applications. Such naturally occurring protein cages, as virus capsids and ferritin cages, serve as excellent templates for functional biomaterials with precise architectures, unattainable by synthetic processes. The regular arrangement of protein subunits within protein cage structures allows for the engineering of specific regions and surfaces of the cage, such as the exterior or interior surfaces. The advantages of using proteins to prepare NPs for drug delivery applications include their abundance in natural sources, biocompatibility, biodegradability, easy synthesis process, and cost-effectiveness. In contrast, other particulate systems such as metallic nanoparticles show several drawbacks including potential toxicity, large size, accumulation, or rapid clearance from the body.

Most requirements for designing an ideal nano-DDS (stability, specificity, or controlled release of the drug) can be acquired using proteins in its structure since protein domains with these functions have been described [15]. As an advantage, proteins can be easily produced in different biological systems [16] such as bacteria [14,17], insect cells [18,19,20], or mammalian cells [21], among others.

In addition, protein-based NPs offer the opportunity for surface modification by standard genetic engineering techniques, or by conjugation of other protein/s and carbohydrate ligands. Protein engineering has been extensively used to redesign structure and function, yielding particles with very narrow size distributions and multiple functionalities. In this context, virus-like particles and other caged protein structures have been explored as nanocarriers for introducing non-native functionalities. This enables targeted delivery to the desired tissue and organ, which further reduces systemic toxicity. Such materials have been developed for applications in the fields of nanotechnology, biotechnology, or drug delivery. The use of protein NPs for such applications could, therefore, prove to be a better alternative to manipulate and improve the pharmacokinetic and pharmacodynamic properties of the various types of drug molecules. Research in this area has been very active for more than two decades, but only in the last years several of these products have been released to the market and are now routinely used in clinics [22].

Protein nano-DDSs can be classified according to the structure resulting from protein interactions in protein nanoboxes, nanoparticles, microspheres, matrices, and fibers. The vast majority of protein nano-DDSs classified as protein boxes have been designed and optimized by nature [23] and have a well-defined structure, divided into external, intermediate, and internal surfaces [24]. Generally, specific ligands of target cells can be bound on the outer surface; the intermediate surface gives stability to the complex; and the internal surface determines which cargo molecules can be introduced [25]. The loaded molecules inside these structures are protected against undesired degradation [23]. Some DDSs, such as viral particles, also have an intrinsic tendency to introduce components into the cell interior [26]. In other cases, they must be modified to incorporate specific peptides and direct them to the target cells. Not being the specific target of this review, these issues (and others) regarding protein-based DDS can be examined in much more detail in previous revisions available in the literature [27,28].

The main interest of protein boxes as nano-DDS is their internal cavity, which will determine the number of molecules that can be internalized. Eukaryotic vaults are protein NPs with a large internal cavity that makes them attractive as a nanocarrier for diverse types of molecules [18,29]. This feature, together with their homogeneity and versatility to be modified and specifically delivered to target cells, make vaults powerful candidates as nano-DDSs.

## 2. Eukaryotic Vaults 

Vaults are ribonuclear-protein cytoplasmic complexes of 13 MDa [19] described for the first time in 1986 [30] as small ovoid bodies similar in structure to the vaults of ecclesiastical buildings (“vaulted ceilings”) [31], by which they were named. Their natural function is not completely elucidated, although several functions related to nuclear transport, immune response [32] and multiresistance in cancer cells [33] have been hypothesized. Although it is well known that they participate in these functions, the mechanism through which it intervenes is not defined. Vault structure is highly conserved among different eukaryotic organisms [20,25,33], which indicates the importance of its functions and the putative biocompatibility that it can present as nano-DDSs [33].

### 2.1. Vaults Structure

The ribonucleoprotein complex is composed of proteins and nucleic acids. The main component is the “major vault protein” (MVP), representing more than 70% of natural vaults [25], while the remaining components are proteins such as poly(ADP-ribose) polymerase (VPARP) and telomerase; and small nontranslated RNA. The recombinant synthesis of MVP monomers is sufficient and allows their spontaneous self-assembling into vaults indistinguishable from natural ones. This has allowed the design, engineering, and production of recombinant vaults [19]. 

**Figure 1:**
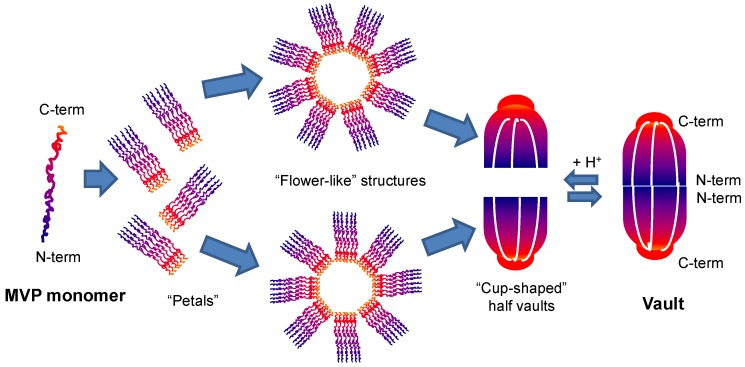
Hierarchical self-assembly of major vault protein (MVP) monomers into closed vaults. Each vault consists in a hollow, barrel-like structure composed of two identical cup-like halves joined at their open ends. Each half vault is in turn composed of a single eight-petaled “flower-like” structure, which is folded into the cup shape. At low pH, the acidic residues at the half-vault interfaces would become neutral, leaving a highly positive charge and inducing the disassembly of the vault particle by charge repulsion. Adapted from [20,36,40,41].

The structure of rat liver vault ribonucleoprotein particles was examined by different staining techniques in conjunction with EM and digestion with hydrolytic enzymes. Quantitative scanning transmission EM demonstrates that each vault particle is a homodimer, composed by two symmetrical halves with a total of 39 copies of MVP in each one [19]. Hydrophobic interactions between MVP domains (the strongest of the structure [18]) direct self-assembling of the vault. Each MVP monomer folds into 12 domains: nine structural repeat domains, a shoulder domain, a cap-helix domain, and a cap-ring domain. Interactions between the 42-turn-long cap-helix domains are key to stabilizing the particle. Freeze-etch revealed that vault can open into flower-like structures, in which eight rectangular petals are joined to a central ring, each by a thin hook. Vaults examined by negative stain and conventional transmission EM (CTEM) confirmed the flower-like structure [34]. The hierarchical self-assembly of MVP monomers into vaults is shown in detail in Figure 1.

The structural arrangement of a single MVP chain into the assembled vault was further analyzed and proven when an ∼9 Å X-ray crystal structure of recombinant vaults purified from insect cells was carried out [35]. A further refinement to 3.5 Å resolution using crystallized rat liver vaults verified the previous low resolution structure prediction [36]. Vault structure analysis by X-ray diffraction at 3.5 Å resolution [25] gives dimensions of approximately 67 × 40 × 40 nm as well as an interior volume of 3.87 × 10^7^ Å^3^. Vaults show a hollow, barrel-shaped structure with two protruding caps and an invaginated waist, based on hierarchical protein self-assembly [36]. 

Moreover, studies based on electron cryotomography showed that intracellular vaults are similar in overall size and shape to purified and recombinant vaults previously analyzed [37]. A 2.1 Å resolution structure of the seven N-terminal repeats (R1–7) of MVP has also been determined [38].

Under physiological conditions there is a balance between the closed and open conformations (separate halves) of vaults, allowing the entry of molecules [39]. An acidification of the medium destabilizes the bonds between monomers, with the exception of the strong hydrophobic joints between the head-helix domains, giving rise to a “flower-like” structure [20,40]. Low-pH condition triggers vault conformational change. Closed intact vaults at pH 6.5 dissociate quickly into half-vaults as the solution pH decrease to less than 4.0 [41]. This dissociation triggered at low pH has been proposed as a useful tool for controlled drug delivery within cellular systems given that endosomes and lysosomes are normally maintained at acidic pH. Thus, this phenomenon is being studied in order to achieve a controlled release of cargo drugs [40]. In this line, Esfandiary et al. employed a variety of spectroscopic techniques (i.e., circular dichroism, fluorescence spectroscopy, and light scattering) along with electron microscopy, to characterize the structural stability of vaults over a wide range of pH (3–8) and temperature (10–90 °C). Ten different conformational states of the vaults were identified over the pH and temperature range studied with the most stable region at pH 6–8 below 40°C and least stable at pH 4–6 above 60 °C [42]. 

Prior crystal structures of the vault have provided clues of its structure but are non-conclusive due to crystal packing. To addres this concern, a recent study determined vaults near-atomic resolution (~4.8 Å) structures in a solution/noncrystalline environment [43]. Authors obtained vaults by engineering at the N-terminus of rat major vault protein (MVP) an HIV-1 Gag protein segment. The barrel-shaped vaults in solution adopt two conformations, 1 and 2, both with D39 symmetry, and comparison with crystallography results shows a major flexible region at the vault shoulder, suggesting that loops near this region could be utilized as peptide fusion sites for engineering purposes. Also in the line to determine vault structure under physiological conditions, Llauró et al. examined the local stiffness of individual vaults and probed their structural stability with atomic force microscopy (AFM) under physiological conditions, showing that the barrel, the central part of the vault, governs both the stiffness and mechanical strength of these particles [44]. In another study, same authors used AFM to monitor the structural evolution of individual vault particles while changing the pH in real time. The results showed that decreasing the pH of the solution destabilize the barrel region, the central part of vault particles, leading to their aggregation. Additional analyses using Quartz-Crystal Microbalance (QCM) and Differential Scanning Fluorimetry (DSF) confirmed AFM experiments [20]. This confirms that low pH weakens the bonds between adjacent proteins.

As described previously, single MVP self-assembly into final vault was modeled using the cryo-EM technique, showing that N-terminal tags were located at the vault waist facing the inside of the particle with longer tags having greater internal density [45]. On the other hand, vaults assembled from MVP containing C-terminal tags displayed extra density at the top and bottom (caps) of the vault, indicating that whereas the N-terminus begins at the inside of the vault waist, MVP C-terminus is exposed at the vault surface [45,46]. The implications of such findings in the putative applications of vault will be discussed in detail in next sections.

### 2.2. Drug Encapsulation within Vaults

**Figure 2:**
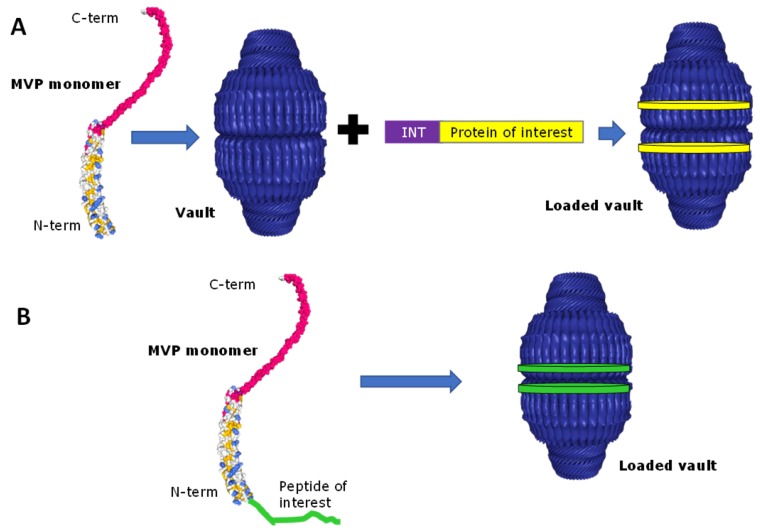
Encapsulation possibilities of recombinant vaults. A. The protein of interest (yellow) is fused to interaction domain (INT) domain (purple). After self-assembly of MVP monomers into vaults, the INT domain will direct loading of cargo protein (shown as two yellow discs) within vaults through specific interaction with MVP monomers. B. The peptide of interest (in green) is expressed as a fusion to N-terminus of MVP monomer. After MVP self-assembly into vaults, cargo peptide will accumulate in the nanoparticle lumen (shown as two green discs). Adapted from [57]. PDB images: 4HL8 Bioassembly 1 and 2.

A key issue in order to apply engineered vaults as an efficient DDS was the development of a procedure to encapsulate foreign materials into the vault lumen. The development of such a strategy relies on previous studies of the VPARP protein, an essential component of native vaults that was identified using the yeast two-hybrid method employing MVP as a bait [47]. With this previous knowledge, a strategy was developed to identify a vault targeting sequence. In this line, structural studies of VPARP and MVP interactions revealed the existence of a domain within the VPARP protein (at its C-terminus, aa 1563–1724), called interaction domain (INT) [48,49,50], which interacts with the inner side of domains 3, 4, and 5 of MVP N-terminal end [51,52]. The fusion of this INT domain at the C-terminus of proteins with therapeutic interest allows their spontaneous encapsulation within vaults without affecting their biological activity, as observed naturally with VPARP. As the INT domain is responsible for binding VPARP to MVP, it was hypothesized to act as a “zip code” directing the protein to the inside of the vault particle (see Figure 2A). This targeting ability was confirmed when the INT domain was fused to proteins with enzymatic or fluorescent activities such as firefly luciferase or a variant of the green fluorescent protein, and coexpressed with MVP in Sf9 insect cells [49], obtaining fluorescently labelled vaults due to cargo protein internalization. INT-tagged proteins copurified with recombinant vaults, and cryo-EM analysis revealed that they were packaged inside the particles into two rings of density, above and below the vault waist. Other proteins with relevant biological activities, such as CCL21 [53], pVI [48], or the antigens MOMP [54] and OVA [55] have been successfully encapsulated by this mechanism. This strategy is depicted in Figure 2. Moreover, this encapsulation process does not not require cotranslation of INT-tagged cargo protein with MVP [39]. INT fusion proteins can be packaged inside recombinant vaults by just mixing them and incubating the mixture on ice for 30 min. This process has been hypothesized to occur via vault “breathing”, a process previously characterized for virus particles. As purified vaults are occasionally observed as half vault structures [41,56], a transient half-vault/whole-vault dynamic could also explain INT protein packaging.

As mentioned before, MVP N-terminus faces the vault lumen, offering an excellent opportunity to encapsulate peptides or proteins. In this context, several fusions have been added to MVP cDNA. The added domain did not interfere in the self-assembly of MVP monomers, rendering vaults similar to native ones, and as expected the new domain were found located inside the nanocages (see Figure 2B). Following this strategy, peptides or proteins like green fluorescen protein [49], a cysteine-rich peptide [45], a His-T7 epitope tag [45], a VSVG epitope tag [45], an adenovirus membrane lytic peptide [48] or an epitope of HIV-1 Gag protein [43] have been successfully encapsulated within vaults.

Compounds not encoded by DNA (a common situation for small molecule drugs) have also been encapsulated within vaults by means of the INT targeting domain [29]. Recombinant INT containing an additional 31 amino acids at the N-terminus including a 6-His tag was used to direct bound species into vaults. This was confirmed with Ni-NTA nanogold, a material with affinity to the 6-His tag. As proof-of-concept, Ni-NTA-nanogold gold clusters were attached to the 6-His tag of three different INT-tagged proteins, forming a Au-INT complex. Then, specific association of gold clusters with the vault was demonstrated by co-immunoprecipitation with agarose beads.

The release rate of proteins encapsulated within vaults by INT-mediated interaction could be slowed down by the substitution of different amino acids of the INT domain by histidines [51]. All these previous results invite to easily visualize that vaults can be in the future an excellent DDS for virtually any peptide or protein with therapeutic potential, just by the simple procedure of fusing them to the INT domain. 

Many chemotherapy drugs are small, hydrophobic molecules that are difficult to administer in aqueous solution and to distribute throughout the body. It is evident that such non-protein molecules cannot be fused to the INT domain, but the internal environment of the vault can be modified to favor its encapsulation. In this line, it has been described that a recombinant nanodisk containing “all trans retinoic acid” (ATRA), a potent but toxic therapeutic compound, could be packaged inside vaults [58]. Such nanodisk was obtained by incubating the fusion of a truncated form of the lipoprotein Apo-AI with INT vault binding domain, together with a dispersion of synthetic phospholipids in aqueous buffer, in the presence of the extremely hydrophobic ATRA drug. Finally, all this complex could be encapsulated within vaults thanks to the presence of the INT domain. Moreover, vault protected the hydrophobic nano-DDS, increasing its half-life and allowing the specific release of the drug. To avoid the previous step of the synthesis of another nano-DDS, recombinant MVPs have been designed that create a more hydrophobic interior environment. Thus, an amphipathic α-helix peptide, originally from the NS5A protein of hepatitis C virus, has been covalently bound to the N-terminal end of MVP [18], without hindering vault self-assembly. This modification has allowed the encapsulation of >2000 molecules (such as bryostatin 1, amphotericin B, or ATRA) per single vault. Bryostatin 1 is of particular therapeutic interest because of its ability to potently induce expression of latent HIV. In this line, results showed that vaults loaded with bryostatin 1 released the drug, resulting in the in vitro activation of latent HIV provirus and induction of CD69 biomarker expression following intravenous injection into mice [18]. This strategy can be of great interest to favor the biodistribution of most hydrophobic drugs.

### 2.3. Vault Targeting and Internalization

**Figure 3:**
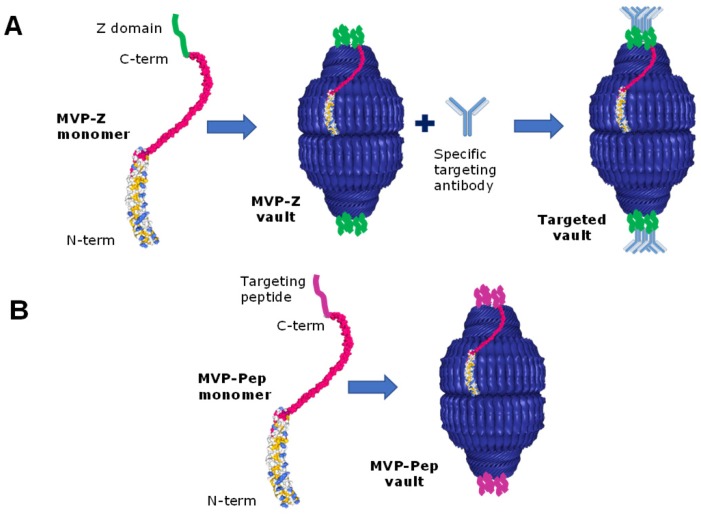
Engineering approaches for targeting vaults. A. The Z domain (in green) is fused to MVP monomer at its C-terminus. After MVP-Z monomer self-assembles into vault, it is incubated with a targeting antibody, that will bind to Z domains through its Fc domain. B. A specific targeting peptide (in purple) is fused to MVP monomer at its C-terminus. After MVP-Pep monomer self-assembles into vault, multiple copies of the targeting peptides will be exposed at the vault’s caps. Adapted from [57]. PDB images: 4HL8 Bioassembly 1 and 2.

To address the ability of vaults to be engulfed and internalized in cells, vaults packaged with INT-tagged fluorescent proteins (GFP or mCherry) were added to HeLa cell cultures and their uptake confirmed by confocal microscopy [46]. This uptake, suggested to occur by endocytosis, was not specific or efficient, and thus considered a positive trend in terms of minimizing putative nonspecific incorporation of nano-DDSs in healthy cells. However, loaded vaults need to be targeted and delivered in a specific way to target cells and tissues. For that purpose, MVP C-terminus has been deeply explored. This C-terminal end faces the outside surface of the vault, and thus it can be modified to incorporate targeting peptides to target and promote specific cellular internalization of vaults into desired cells. In the frame of this strategy, the ability to specifically target the vault will rely and depend on the ability to find receptors specifically expressed (or clearly overexpressed in comparison with healthy cells) and the obtention of specific ligands recognizing such specific receptors in the target cell. Then, such ligands should be fused to the vault surface in order to recognize their target. For example, since epidermal growth factor (EGF) is upregulated in numerous cancer cell types, tags have been engineered and tested as fusions to the C-terminus of MVP to facilitate targeting to epithelial cancer cells via the EGF receptor (EGFR): a 33 amino acid Fc-binding peptide (called the Z domain) and the 55 amino acid epidermal growth factor (EGF). The modified vaults bound specifically to cancer cells either directly (EGF modified vaults) or as mediated by a monoclonal antibody (anti-EGFR) bound to recombinant vaults containing the Z domain (see Figure 3A). The EGF modified vaults have the ability to specifically bind cell surface receptors and trigger receptor activation in a manner similar to recombinant human EGF [46]. Thus both specific (peptide-directed) and general (antibody-mediated) methods could be used to target recombinant vault particles to cells, representing an essential advance towards the use of recombinant vaults as targeted DDSs.

Since recombinant vaults will expose one copy of the targeting peptide for each copy of engineered MVP monomer (see Figure 3B), the size of the peptide or protein fused to the C-terminal end of the MVP plays an important role in the stability of the vaults. The fusion of epidermal growth factor (EGF) [46], whose receptor is overexpressed in several cancers has been studied. It was observed that the presence of EGF in all the copies of MVP produces instability, interfering in its structuring, finally rendering insoluble vaults. To solve this problem, vectors were designed with two promoters that allow, on the one hand, the expression of MVP associated with EGF; and on the other, the expression of natural MVP [46]. This reduced the number of EGF present in each vault, allowing its correct structure, solubility, and ability to reach target cells. 

The main route of administration of vaults consists of intratumoral injection [53], while the intranasal route has been studied for the use of vaults as vaccines [54]. Oral administration is hampered by the structural changes that vaults may undergo when exposed to acidification [20,40] that occurs in the gastrointestinal tract, and vaults specifically designed to cross the blood–brain barrier has not been developed yet. 

Vaults specifically directed to target cells by ligands or antibodies will enter the cell interior by endocytosis [48,53], following the endosomal pathway until its degradation in lysosomes. Its success as nano-DDS depends on its ability to release the therapeutic components to the cytoplasm of the cells and not be degraded in the lysosomes. In order to avoid the degradative pathway and increase the release of the drug to the cytoplasm, the fusion of the lytic domain of the pVI protein of the adenovirus to the N-terminal end of the MVP has been studied [48]. It was demonstrated that pVI-vaults could disrupt the endosomal membrane using three different experimental protocols including enhancement of DNA transfection, codelivery of a cytosolic ribotoxin, and direct visualization by fluorescence. The early exit of the lysosome occurs without causing nonspecific damage to the cell, although it has been observed that a high concentration of this peptide can result in unwanted cellular apoptosis [48]. However, therapeutic effect greatly depends on the efficient release of the cargo protein from the DDS. In this context, it is hypothesized that the release rate of the cargo from the vault lumen is directly related to the interaction between MVP and INT domain. To further explore the release of molecular cargos from the vault nanoparticles, the interactions between isolated INT-interacting MVP domains (iMVP) and wild type INT has been determined and compared to two structurally modified INTs: first, a 15-amino acid deletion at the C terminus (INTΔC15) and, second, a histidine substitution at the interaction surface (INT/DSA/3 H) to impart a pH-sensitive response [51]. The introduction of histidines to His-INT resulted in stronger interaction between His-iMVP and His-INT/DSA/3 H compared to the wild type His-INT at both pH 6.0 and 7.4. This study implies that modulation of molecular release rate from the vault is possible by tuning the proportion of wild type and histidine-substituted INT or by truncation of the INT domain.

The above-mentioned strategies are limited by the capability to genetically engineer MVP protein to fuse the desired targeting peptide to it and therefore, to the resulting vault. Recently, a different approach has been proposed based on covalent chemical modifications of MVP residues. As other protein-based supra-macromolecular structures, vaults contain many derivatizable amino acid side chains. The new approach [19] was focused on establishing the comparative selectivity and efficiency of chemically modifying vault lysine and cysteine residues, using Michael additions, nucleophilic substitutions, and disulfide exchange reactions. Given the great number of vault lysine residues and the versatile chemistry of thiols, authors demonstrated a simple, robust technique to efficiently convert these more abundant residues into thiol terminated side chains. Using such chemistry, vaults doubly modified with a fluorescein reporter probe and cell-penetrating octaarginine peptides attached via a redox-sensitive cleavable or noncleavable linker were obtained. Relative to unmodified vaults, the resultant modified vaults showed no adverse particle effects following chemical modification while clearly demonstrating increased cellular uptake into cells of interest. This study provides a chemical foundation for predictable and fast vault modification, as required for the use of engineered vaults in imaging, therapeutic delivery, or basic biological research.

## 3. Recombinant Vaults Production and Purification

The baculovirus–insect cell system is today one of the most commonly used strategies to produce recombinant proteins. Since insect cells are one of the few eukaryotes lacking endogenous vaults, they have been the standard cell factory to obtain recombinant vaults. The current production of recombinant vault nanoparticles is mainly performed in *Spodoptera frugiperda* (Sf9) insect cells [59], where expression of only the MVP protein can direct the assembly of vault-like particles on polyribosomes [59,60]. However, this approach is complex and costly for industrial scale applications. The construction of a recombinant baculovirus containing a gene of interest requires a tedious and time-consuming (3–6 months) process. After that, routine growing, titration, and maintenance of the baculovirus stocks are also required. Moreover, continuous protein production is hampered by insect cells lysis during infection. Release of intracellular proteins from lysed cells, or removal or inactivation of progeny baculoviruses released by budding off from infected cells, may result in protein degradation by proteases and may also complicate downstream process [61,62,63].

To date, one significant change in the process has been the proposal of the replacement of the sf9 insect cells for insect larvae, which allows a greater production. Protein expression levels in baculovirus-infected larvae can be very high, reducing costs for large-scale production. In this line, a procedure was reported, based in baculovirus-infected insect larvae as starting material [40]. Nevertheless, due to general unfamiliarity with larval systems and restricted or low access to cell culture facilities to any research laboratory, this approach has not gained widespread popularity in most molecular biology laboratories in North America and Europe.

According to all these drawbacks related to expression systems, there is the need to develop alternative cell factories for recombinant vault production. Among the available organisms, yeast is a promising one for large-scale expression and preparation for human applications. Yeast have been successfully used in the last decades for recombinant protein production [64] and, for the synthesis of protein nanostructures such as virus-like particles [65], are similar in size and structure to vaults. In this context, it has been recently described for the first time the production of vaults in the yeast *Pichia pastoris* [66]. Expression of MVP alone in *P. pastoris* led to the formation of intact vaults, morphologically similar to endogenous vaults isolated from other eukaryotes. Moreover, such yeast vaults retained the ability to interact with INT-fused proteins, revealing *P. pastoris* as a new, promising alternative to insect cells for producing recombinant vaults.

To develop an economically competitive platform based on recombinant vaults, efficient downstream processes also need to be set-up and optimized. The interest of its use as nano-DDS requires the optimization of the process in order to increase the scale and yield [53] of production. The high size of the vault ribonucleoprotein complex complicates its downstream. For its purification, sucrose gradients [59,67], and continuous ultracentrifugation steps [57] have been used in a traditional manner, procedures rather complex and labor intensive. This technique greatly hinders large-scale production [57], both for the time devoted to achieving the needed high purity and the small amount obtained. Recombinant MVP is purified from insect cell extracts in a procedure requiring three ultracentrifugation steps and two additional gradient centrifugations. Sucrose or cesium chloride gradient ultracentrifugation [68,69] is generally considered to be chemically and physically appropriate for purification of different protein-based nanoparticles, including VLPs, but this general approach is labor-intensive, time-consuming, and scale-restricted [70], and can be associated with unexpected batch-to-batch variation. Although several reports have shown that gradient ultracentrifugation could be employed to purify VLPs, it provided only low yield and failed to remove impurities (including recombinant baculoviruses) from the final products [71].

Taking all these facts together, the need for the development of faster and easier downstream procedures for recombinant vaults is clear, and many efforts are being devoted to such purpose. In this line, efforts have been made in the substitution of centrifugation by special chromatographic columns, which significantly reduce the purification time. According to this approach, after removing cell debris, clarified lysate is loaded into an ion exchange column for large particles (Fractogel^®^ EMD TMAE) and then into a gel filtration column, rendering final overall purities higher than 99% [33,40]. More recently, a two-step protocol for vault purification has been described, based on dialysis step and size-exclusion chromatography [72]. In this work, vaults were purified by a first dialysis step using a 1 MDa molecular weight cutoff membrane and a subsequent size-exclusion chromatography (SEC) on a Sepharose CL-6B column, rendering vaults with 90–95% purity and yields of 15 mg protein from 0.7–0.8 g cell samples pelleted from 50 mL of culture medium. Despite all these efforts and advances, vaults purification is still performed basically by the original protocol based on several ultracentrifugations. 

In Table 1 it is shown the currently used expression systems and purification procedures for the obtention of recombinant vaults. Apart from those already in use, large scale and reproducible vault particle purification methodologies will be needed, preferably without ultracentrifugation steps. Thus, it is not difficult to foresee that new, improved purification methods will surely appear in the future that will allow a fast, cost-efficient vault purification. 

## 4. Vaults Applications in Nanomedicine and Clinical Trials

As a naturally occurring nanocage, vault is a promising candidate for DDS to target and deliver therapeutic molecules to damaged cells or tissues. Being highly stable structures in vitro, it is reasonable to hypothesize that vaults will be also stable in the bloodstream. However, putative immunogenic reactions from the body to recombinant vaults could restrict their application in clinical trials, mainly when using repetitive administrations. Several studies indicate that vaults are nonimmunogenic. For example, it was not possible to elicit antibodies in rabbits against purified vaults, and an antigenic response could only be induced when vaults were hemocyanin cross-linked prior to injection into rabbits [30]. In another study, the immunobistocbemical expression of MVP protein in freshly frozen normal human tissues and in 174 cancer specimens of 28 tumor types was analyzed, showing a broad distribution of MVP in both normal and tumoral human tissues [73]. Finally, immunogenicity of recombinant vaults in rats using subcutaneous administration showed no immunoreactivity against the recombinant vaults [57]. All these data suggest that, being ubiquitous throughout the human body, vaults are bio-invisible and nonimmunogenic to the human immune system. 

To date, it is the American company Vault Pharma, a spin-off of the research group, that led by Dr. Leonard Rome (Dept. of Biological Chemistry, UCLA) discovered vaults in 1986, which holds intellectual property over vaults and some of their applications by means of several patents and, therefore, which is developing vaults as new DDS, some of them already in clinical trials. Such patents cover aspects including recombinant production, and vaults as carriers for biomolecules (such as cytokines and hydrophobic molecules) delivery. The vault is being explored and used as a tool to modulate the activity of the immune system. Vault Pharma’s technology platform uses the vault particle to deliver peptides for unique immune signaling, exploiting the vault natural function as an immunological signal alert progenitor (characterized by vault being rapidly ingested by antigen presenting cells (APCs), specifically macrophages and dendritic cells). It was described that vaults are an early alert signal to the immune system once a cell is lysed and vaults are released to the extracellular space where they are rapidly engulfed by APCs. In this sense, it has been described that dendritic cells efficiently internalize vault nanocapules [54]. Therefore, current clinical trials based on these properties takes advantage of this natural property of vaults to trigger an immune response that is noninflammatory and results in many propitious effects including stimulation of extraordinarily high levels of antigen specific CD4 and CD8 T cells. The projects developed can be classified as oncological immunology and immunological activation to prevent infections.

For the treatment of oncology malignancies, vaults are designed to modulate the immune system, so it can slow or stop tumor development. The most advanced design consists of vaults (not specifically targeted) containing the CCL21 chemokine. This chemokine CCL21 is expressed mainly in the high venules of the lymph nodes and Peyer’s patches [53] and act as a chemoattractant of cells of the immune system that express the CCR7 receptor. The strategy, which has shown its effectiveness, consists of directing the cells of the immune system to the tumor zone to stop the growth of the tumor [53]. The encapsulation of chemokine CCL21 occurs by fusion of the INT [53] domain at the C-terminus of CCL21. The MVP monomer is not fused to peptides ligands or specific antibodies, so the intratumoral administration is the only possible to act in a specific way. This explains that the use of the same nano-DDS is being studied to treat different types of cancer. The most advanced clinical trial that uses the strategy mentioned is directed against lung cancer. In the preclinical phase, the effectiveness of intratumoral administration of vaults loaded with CCL21 in mouse models was demonstrated [33,53]. At these localized sites there is an increase in the activity of the T lymphocytes against the tumor cells which results in the inhibition of their growth. Currently, the project is at the beginning of the development phase I. As future perspectives, the fusion of different ligands or antibodies specific for tumor cells to CCL21-vaults would allow intravenous administration and the possibility of reaching difficult-to-access tumors [74].

Other techniques of activation of the immune system have been used to stop the tumor development. Different cytokines (IL7 and CCL19) [75] have been encapsulated in vault nanoparticles and their effect on tumors is being tested. Another route that is being developed is the encapsulation of antigens characteristic of tumors such as NY-ESO [76], acting the vault as if it were a vaccine, activating the immune system to recognize tumor cells and stop their growth.

On the other hand, the approach to control infectious diseases relies in the controlled encapsulation of specific antigens of certain pathogenic microorganisms (Chlamydia, HIV, Influenza, HPV y Burkholderia), allowing an optimal activation of the immune system by the slow release of immunogenic epitopes from the vault. For example, it has been explored the ability (as vaccines for *Chlamydia trachomatis*) of engineered vaults containing an immunogenic epitope of this pathogen, the polymorphic membrane protein G (PmpG), to be internalized into human monocytes and behave as a “natural adjuvant”. Such PmpG-1-vaults were able to activate caspase-1 and to stimulate IL-1β secretion, and immunization of mice with PmpG-1-vaults induced PmpG-1 responsive CD4(+) cells upon restimulation with PmpG peptide in vitro [77]. In another approach to fighting Chlamydia infections [54], the immunogenic protein major outer membrane protein (MOMP) of *Chlamydia muridarum* was encapsulated within hollow, vault nanocapsules (MOMP-vaults) that were engineered to bind IgG for enhanced immunity. Intranasal immunization with such constructions induced anti-chlamydial immunity plus a significantly attenuated bacterial burden following challenge infection. Based on all these backgrounds, Vault Pharma is conducting several clinical trials against different infections, being the most advanced of such trials in their preclinical phases and alert the immune system against Chlamydia, HIV and Influenza pathogens. A brief list of such trials is shown in Table 2. 

However, transfer of vaults use to the clinic presents different challenges to overcome. First, current protocols for the production and purification of vaults need to be optimized. Each recombinant vault requires the design, construction and maintenance of a baculovirus vector, a tedious and time-consuming process. In this line, new expression systems (mainly insect larva or yeast cells) are emerging as new actors in the field, with promising (but still preliminary) results. Also, downstream processeses (based on tedious and time-consuming ultracentrifugation steps) need to be optimized. 

On the other hand, administration routes for vault-based nanomedicines is another issue that has been explored in detail. Oral administration of vaults—the preferred route of administration in the pharmaceutical industry—presents a clear problem due to the structural changes suffered by the vault in acidic environments such as the stomach. Thus, for this case, vaults would need to be strongly engineered and modified to overcome such barrier. To date, the design of vaults to cross the blood–brain barrier has not been developed. However, the natural presence of vaults in the central nervous system [78] has been identified, suggesting that with the right ligand, vaults could cross the blood–brain barrier without causing damage to the central nervous system. Intranasal administration has also been explored for vault-based nanomedicines containing immunogenic proteins, successfully acting as “smart adjuvants” inducing protective immunity at distant mucosal surfaces while avoiding inflammation. Current clinical trials are focused on the treatment of different tumors by vaults loaded with molecules boosting the immune system. In this case, vaults are locally (intratumoral) administered, thus not taking advantage of the possibility to engineer vaults to specifically target them. This may be due to the interest of obtaining a generalized product for different tumors. The use of targeted and engineered vaults will surely expand the portfolio of available therapies for malignancies and other pathologies in the next years. However, and as far as we know, the immunogenicity of recombinant vaults using other administration routes (as intraperitoneal or intravenous) has not been tested yet.

## 5. Conclusions and Future Perspectives

Vaults are naturally occurring nanoparticles found widely in eukaryotic cells. They can be produced in recombinant expression systems in large quantities by expressing the MVP protein monomer, which spontaneously self-assembles to originate the final barrel structure. Vaults have been proposed as a new nano-DDS able to improve the efficacy and reduce the side effects of current treatments since they show the characteristics of an ideal nano-DDS: biocompatibility, encapsulation capacity of hydrophobic and hydrophilic molecules, controlled release, and specific targeting, among other features. Its large internal cavity allows the encapsulation of large quantities of molecules of interest. Vaults are a versatile system, in which encapsulation of cargo proteins is achieved by the fusion of the INT domain to the C-terminal end of the therapeutic protein. Non-protein cargo molecules can also be charged inside by modifying the internal cavity of the vault. Moreover, modification of MVP C-terminal end by the fusion of appropriate targeting peptides allows vault specific targeting to cells and organs.

Despite the great potential shown by vaults as smart DDSs, research related to these protein-based nanoparticles has not been blooming as expected for such promising DDS. As mentioned before, a single company holds the intellectual property of vaults through numerous patents covering several aspects related to their production and exploitation. Such protection might represent a factor hampering the further study of vaults by other research groups. However, and despite this potentially discouraging fact, other laboratories are also devoting efforts to the study of different aspects of vaults, from more basic and structural characterization, to their application in fields other than cancer or infectious diseases treatment, confirming vaults as one of the most promising protein-based DDS.

Finally, it is worthy to note that vaults appear at present day as a promising tool not only in the nanomedicine field, but also in another biotechnological applications. A preliminary study [79] showed the development of efficient and sustainable vault-based bioremediation approaches for removing multiple contaminants, such as phenol, from drinking water and groundwater, using vaults loaded with the enzyme manganese peroxidase [80]. In this line, vaults have been recently proposed as a highly efficient system to immobilize enzymes (“nanosupported” enzymes with biodegradative or antimicrobial activities) with potential use as portable water treatment technologies [80]. This will surely expand the opportunities and applications of these protein-based, self-assembled nanoparticle named vaults.

## Figures and Tables

**Table 1:** Expression systems and purification protocols currently used in the manufacturing of recombinant vaults.

Expression System [Ref.]	Purification Method	Final Yield	Advantages	Disadvantages
Baculovirus infection on sf9 cells [59]	Several saccharose gradient ultra-centrifugations	~10 mg/L	No endogenous vaults	Time-consuming, tedious downstream process
Baculovirus infection on sf21 cells [72]	Dialysis and size-exclusion chromatography	~0.5–1 mg/L	No endogenous vaults Quick, easy and cheap downstream process	Low yields
Baculovirus oral infection on insect larvae [40]	Ion exchange (cationic) and size-exclusion chromatography	Up to several grams	No endogenous vaults High yields	Difficult scale-up Slow production rate
Yeast cells (*Pichia pastoris*) [66]	Sucrose gradient ultracentrifugation and ion exchange (anionic) chromatography	~7–11 mg/L	No endogenous vaults Fast and cost-efficient production	Need scale-up

**Table 2:** Current vaults applications in nanomedicine. Specific applications are classified according to the stage of their respective clinical trials. Source: https://vaultpharma.com/pipeline/.

Vaults Application	Clinical Trial Stage		
	Drug Discovery	Preclinical	Phase I
Oncology immunology	Pancreatic cancer Prostate cancer Head and neck cancer Renal cancer Bladder cancer Colon cancer Breast cancer Graft vs host disease Pulmonary fibrosis Solid tumors	Lung cancer Melanoma Glioblastoma	Lung cancer
Immunological activation for infection prevention	HPV cancer Burkholderia	Chlamydia HIV/AIDS Influenza	
